# Supplementary material for: Diagnosis, Management and Prescription Practices of Adrenaline in Children with Food-Induced Anaphylaxis: Audit in a Specialized Pediatric Allergy Department
Source: J Pers Med. 2022 Sep 9;12(9):1477. doi: 10.3390/jpm12091477 (PMC9501412; doi:10.3390/jpm12091477)
Supplement: Supplementary file 1 [file jpm-12-01477-s001.zip › Supplementary Materials S2.pdf]

PEDIATRIC FOOD-INDUCED ANAPHYLAXIS HISTORY FORM

Date : …………………..………..

Medical File Number :

Name - Surname :

Gender : ◯ Male **/** ◯ Female

Date of Birth :

Address :

Telephone Number :

E-mail :

Attending Physician :

**Culprit Food Allergen**	Dairy ◯ Eggs ◯ Wheat ◯ Peanut ◯ Nuts ◯ Fish ◯ Seafood ◯ Other :	
**Symptoms Recording per System**		
**Sudden onset of skin symptoms** *(pruritus or erythema)*	YES ◯	NO ◯
**Sudden onset of angioedema** *(lips or tongue)*	YES ◯	NO ◯
**Upper respiratory system symptoms** *(sudden onset rhinorrhea, sneezing, hoarseness, "barky" cough, throat tightness)*	YES ◯	NO ◯
**Lower respiratory system symptoms** *(shortness of breath / dyspnea, wheezing, cyanosis)*	YES ◯	NO ◯
**Blood pressure drop** *(hypotension, dizziness, syncope)*	YES ◯	NO ◯
**Persistent gastrointestinal symptoms** *(persistent abdominal pain, vomiting)*	YES ◯	NO ◯
**Vital Signs** *(during the episode)*		
**Blood Pressure** *mmHg*	**SatO$_2$** *%*	**Heart Rate** */min*
**Anaphylaxis Diagnosis** *(based on EAACI's definition)*	YES ◯	NO ◯
**Grade** *(based on Sampson's criteria)*	1 ◯ 2 ◯ 3 ◯ 4 ◯ 5 ◯	
**Medications for future episode prescription**		
**Adrenaline**	YES ◯	NO ◯
**Antihistamine**	YES ◯	NO ◯
**Cortisone**	YES ◯	NO ◯
**β_2-agonist**	YES ◯	NO ◯
