# Supplementary material for: Diagnosis, Management and Prescription Practices of Adrenaline in Children with Food-Induced Anaphylaxis: Audit in a Specialized Pediatric Allergy Department
Source: J Pers Med. 2022 Sep 9;12(9):1477. doi: 10.3390/jpm12091477 (PMC9501412; doi:10.3390/jpm12091477)
Supplement: Supplementary file 1 [file jpm-12-01477-s001.zip › Supplementary Table S1.pdf]

Table S1: Grading of Food-Induced Anaphylaxis According to Severity of Clinical Symptoms†

GRADE	SKIN	GASTRO-INTESTINAL	RESPIRATORY	CARDIOVASCULAR	NEUROLOGICAL
1	Localized pruritus, flushing, urticaria, angioedema	Oral pruritus, oral "tingling," mild lip swelling			
2	Generalized pruritus, flushing, urticaria, angioedema	Any of the above, nausea and/or emesis x's 1	Nasal congestion and/or sneezing		Change in activity level
3	Any of the above	Any of the above plus repetitive vomiting	Rhinorrhea, marked congestion, sensation of throat pruritus or tightness	Tachycardia (increase >15 beats/min)	Change in activity level plus anxiety
4	Any of the above	Any of the above plus diarrhea	Any of the above, hoarseness, "barky" cough, difficulty swallowing, dyspnea, wheezing, cyanosis	Any of the above, dysrhythmia and/or mild hypotension	"Light headedness," feeling of "pending doom"
5	Any of the above	Any of the above, loss of bowel control	Any of the above, respiratory arrest	Severe bradycardia and/or hypotension or cardiac arrest	Loss of consciousness

†. Sampson, H.A. Anaphylaxis and emergency treatment. *Pediatrics* **2003**, *111* (Pt 3), 1601–1608. Available online: https://pubmed.ncbi.nlm.nih.gov/12777599/ (accessed on 14 April 2022).
